# Genetic and molecular features of seizure-freedom following surgical resections for focal epilepsy: A pilot study

**DOI:** 10.3389/fneur.2022.942643

**Published:** 2022-09-16

**Authors:** Shreya Louis, Robyn M. Busch, Dennis Lal, Jennifer Hockings, Olivia Hogue, Marcia Morita-Sherman, Deborah Vegh, Imad Najm, Chaitali Ghosh, Peter Bazeley, Charis Eng, Lara Jehi, Daniel M. Rotroff

**Affiliations:** ^1^Cleveland Clinic Lerner College of Medicine, Cleveland Clinic, Cleveland, OH, United States; ^2^Epilepsy Center, Neurological Institute, Cleveland Clinic, Cleveland, OH, United States; ^3^Genomic Medicine Institute, Lerner Research Institute, Cleveland Clinic, Cleveland, OH, United States; ^4^Center for Personalized Genetic Healthcare, Community Care and Population Health, Cleveland Clinic, Cleveland, OH, United States; ^5^Department of Pharmacy, Cleveland Clinic, Cleveland, OH, United States; ^6^Department of Quantitative Health Sciences, Lerner Research Institute, Cleveland Clinic, Cleveland, OH, United States; ^7^Department of Biomedical Engineering, Lerner Research Institute, Cleveland Clinic, Cleveland, OH, United States; ^8^Department of Genetics and Genome Sciences, Case Western Reserve University School of Medicine, Cleveland, OH, United States; ^9^Endocrinology and Metabolism Institute, Cleveland Clinic, Cleveland, OH, United States

**Keywords:** epilepsy, resection, genetics, seizure-freedom, prediction, surgical outcomes, genetic variant

## Abstract

**Objective:**

Seizure outcomes after brain surgery for drug-resistant epilepsy (DRE) are very heterogeneous and difficult to predict with models utilizing the current clinical, imaging, and electrophysiological variables. In this pilot study, we investigated whether genetic and molecular biomarkers (e.g., genomic, transcriptomic) can provide additional insight into differential response to surgery.

**Methods:**

Post-operative seizure-outcomes were collected at last follow-up (>6 months) for 201 adult patients with DRE who underwent surgery between 2004 and 2020. Resected tissue was sent for miRNA sequencing (*n* = 132) and mRNA sequencing (*n* = 135). Following the selection of 10 genes (*SCN1A, NBEA, PTEN, GABRA1, LGL1, DEPDC5, IL1A, ABCB1, C3, CALHM1*), we investigated SNPs in those 10 genes from previously acquired exome sequencing data (*n* = 106). Logistic regression was performed to test for associations between individual features (mRNAs, miRNAs, and SNPs) and post-operative seizure-outcome with an exploratory FDR *P* < 0.25 as the threshold for significance. Post-operative time-to-seizure analyses were performed for each SNP using a Cox proportional hazards model.

**Results:**

The majority of patients (83%) had temporal lobe epilepsy. Mean age at surgery was 38.3 years, and 56% were female. Three SNPs (rs10276036, rs11975994, rs1128503) in multi-drug resistance gene, *ABCB1*, were associated with post-operative seizure outcomes. Patients with alternate alleles in *ABCB1* were more likely to be seizure-free at last follow-up (52–56% reduction in seizure recurrence; FDR *P* = 0.24). All three SNPs were in linkage disequilibrium and highly correlated with each other. Median post-operative time-to-seizure was 63 months for patients with 2 alternate alleles, 24–33 months with 1 alternate allele, and 10–11 months with 0 alternate alleles. These SNPs improved outcome prediction beyond MRI and sex alone. No independent miRNAs or mRNAs were significantly associated with seizure-outcome (*P* > 0.05). However, pathway analysis identified “cancer drug resistance by drug efflux” (mir-154 and mir-379) as enriched (*P* = 0.02), supporting the role of drug response genes in post-operative seizure recurrence.

**Significance:**

*ABCB1* may have a role in epileptogenesis and surgery outcomes independent of its drug efflux activity necessitating further investigation. SNPs in *ABCB1* may serve as independent predictors of post-operative outcome.

## Introduction

The use of genetics for clinical prediction of neurological diseases is growing, and genetic testing is now widely applied in neuro-oncology to predict tumor responsiveness (1) and in hereditary movement disorders, such as Huntington disease (2). Within the field of epilepsy, the utility of genetic testing has been predominantly explored in pediatric epilepsy syndromes (3), neurodevelopmental disorders with epilepsy (4), and heritable forms of epilepsy (5), with the overarching goal of improving diagnosis and potentially directing therapy. However, robust evidence regarding the value of genetic [e.g., single nucleotide polymorphisms (SNPs)] and other molecular markers (e.g., non-coding RNAs, coding mRNAs) to guide epilepsy surgical treatment decisions is lacking and often limited to small case series (6).

Approximately one percent of the United States population has epilepsy, and one-third of these patients have drug-resistant epilepsy (DRE). When safe and possible, surgical resection is the treatment of choice for patients with DRE. Selection of appropriate surgical candidates currently involves a multidisciplinary committee of radiologists, neurologists, and neurosurgeons using a variety of diagnostic and clinical information (e.g., magnetic resonance imaging (MRI), electroencephalography (EEG) video monitoring during inpatient stays, seizure semiology, invasive EEG monitoring) (7). Previously developed statistical models predominantly use clinical, imaging, and electrophysiological variables to predict seizure-freedom and cognitive-decline post-operatively (8–10).

For example, a nomogram using age of seizure onset, age at surgery, seizure frequency, sex, presence of generalized tonic-clonic seizures, and pathology of underlying seizures achieved 60% discrimination (C-statistic = 0.6) to predict seizure freedom at 2 and 5 years post- operatively in DRE patients (8). The inclusion of MRI and epilepsy duration improved discrimination of seizure outcomes to 65% in patients with temporal lobe epilepsy (10), and incorporation of quantitative MRI volumetrics further improved prediction accuracy to 73% (11).

While these advancements have improved the ability to predict patient responses to surgical resection, post-operative seizure outcomes are still heterogeneous, warranting further investigation to identify additional clinical predictors.

Our team has proposed a mechanistic framework for post-operative seizure outcomes, in which incomplete resection or inaccurate localization of the epileptogenic zone contributes to early seizure recurrence after surgery (*early* surgical failures). We hypothesize that *late* surgical failure may be in part due to underlying molecular or genetic mechanisms that create and maintain epileptogenic zones even after the initial epileptogenic tissue was resected. Preliminary studies have explored associations between transcript expression in the resected tissue and late seizure recurrence post-operatively (12), suggesting alterations in neuroinflammatory and brain healing/remodeling pathways. In this study, we investigate whether germline genetic variation, tissue messenger RNA (mRNA) expression, and tissue microRNA (miRNA) expression are associated with post-operative seizure-freedom in a well-characterized cohort of patients with DRE. The goal is to better understand the underlying mechanisms of seizure response and the potential for molecular-based predictions of seizure-freedom following surgical resections.

## Methods

### Cohort selection and demographics

Our cohort consisted of adult patients with DRE who underwent surgical resection at the Cleveland Clinic from 2004 to 2020 and were enrolled in the Cleveland Clinic Epilepsy Center Biorepository (allowing for DNA and RNA studies on brain and blood biospecimens; IRB #12-1000) or the Epilepsy Surgery Nomogram protocols (allowing for DNA analyses only; IRB #16-1539). At least 6 months of follow-up after resection was required. Patients were excluded if they had benign or malignant brain tumors or vascular malformations within the region of resection. All specimen and data collection were approved by the Cleveland Clinic institutional review board and obtained with informed patient consent. DNA sequence data was available for 106 patients, miRNA sequencing data from resected tissue was available for 132 patients, and mRNA sequencing from the resected brain tissue was available for 135 patients. Detailed patient characteristics can be found in Table 1.

**Table 1:** Cohort characteristics.

**Overall cohort**	**Seizure-free** **(*N* = 105)**	**Recurrent** **(*N* = 96)**	**Total** **(*N* = 201)**
**Age at surgery (years), mean (SD)**	38.9 (14.7)	37.6 (14.9)	38.3 (14.8)
**Epilepsy resection sub-type**			
Temporal (%)	90 (85.7)	80 (83.3)	167 (83.1)
Extratemporal (%)	15 (14.3)	19 (19.8)	34 (16.9)
Frontal (%)	13 (86.7)	13 (68.4)	26 (76.5)
Parietal (%)	2 (13.3)	3 (15.8)	5 (5.9)
Occipital (%)	0 (0.0)	1 (7.7)	1 (2.9)
Multi-lobar (%)	0 (0.0)	2 (10.5)	2 (5.9)
Female sex (%)	61 (58.1)	51(53.1)	112 (55.7)
**Surgical pathology**			
Mesial temporal sclerosis (MTS) (%)	14 (13.3)	10 (10.4)	24 (5.0)
Malformations of cortical development (MCD) (%)	46 (43.8)	47 (49.0)	93 (46.3)
Cryptogenic (%)	6 (4.7)	4 (41.7)	10 (12.0)
MTS and MCD (%)	23 (21.9)	14 (14.6)	37 (18.4)
Other (%)	16 (15.2)	21 (21.9)	37 (18.4)
White race (%)	–	–	201 (100)
**Exome data cohort**	**Seizure-free** **(*****N*** = **47)**	**Recurrent** **(*****N*** = **59)**	**Total** **(*****N*** = **106)**
Age at surgery (years), mean (SD)	36.6 (14.8)	35.3 (15.0)	35.6 (13.7)
**Epilepsy resection sub-type**			
Temporal (%)	36 (76.6)	43 (72.9)	79 (74.5)
Extratemporal (%)	11 (23.4)	16 (27.1)	27 (25.5)
Frontal (%)	9 (81.8)	11 (68.8)	20 (74.1)
Parietal (%)	2 (18.2)	3 (18.8)	5 (18.5)
Multi-lobar (%)	0 (0.0)	2 (12.5)	2 (7.4)
Female sex (%)	28 (59.6)	30 (50.8)	58 (54.7)
**Surgical pathology**			
Mesial temporal sclerosis (MTS)	6 (12.8)	3 (5.1)	9 (8.5)
Malformations of cortical development (MCD)	22 (46.8)	33 (55.9)	55 (51.9)
Cryptogenic	3 (6.4)	2 (3.4)	5 (4.7)
MTS and MCD	7 (14.9)	6 (10.2)	13 (12.3)
Other	9 (19.1)	15 (25.4)	24 (22.6)
**Tissue mRNA cohort**	**Seizure-free** **(*****N*** = **74)**	**Recurrent** **(*****N*** = **61)**	**Total** **(*****N*** = **135)**
Age at surgery (years), mean (SD)	40.3 (14.1)	39.3 (14.4)	39.9 (14.2)
**Epilepsy resection sub-type**			
Temporal (%)	67 (90.5)	54 (88.5)	121 (89.6)
Extratemporal (%)	7 (9.5)	7 (11.5)	14 (10.4)
Frontal (%)	6 (85.7)	4 (57.1)	10 (71.4)
Parietal (%)	1 (14.3)	0 (0.0)	1 (7.1)
Occipital (%)	0 (0.0)	1 (14.3)	1 (7.1)
Multi-lobar (%)	0 (0.0)	2 (28.6)	2 (14.3)
Female sex (%)	43 (58.1)	32 (52.5)	75 (55.6)
**Surgical pathology**			
Mesial temporal sclerosis (MTS)	10 (13.5)	8 (13.1)	18 (13.3)
Malformations of cortical development (MCD)	31 (41.9)	30 (49.2)	61 (45.2)
Cryptogenic	5 (6.8)	3 (4.9)	8 (5.9)
MTS and MCD	19 (25.7)	10 (16.4)	29 (21.5)
Other	9 (12.2)	10 (16.4)	19 (14.1)
**Tissue miRNA cohort**	**Seizure-free** **(*****N*** = **75)**	**Recurrent** **(*****N*** = **57)**	**Total** **(*****N*** = **132)**
Age at surgery (years), mean (SD)	40.3 (14.0)	38.4 (14.4)	39.5 (14.2)
**Epilepsy resection sub-type**			
Temporal (%)	68 (91.0)	51 (89.5)	119 (90.2)
Extratemporal (%)	7 (8.9)	6 (10.5)	13 (9.8)
Frontal (%)	6 (85.7)	3 (50.0)	9 (69.2)
Parietal (%)	1 (14.3)	0 (0.0)	1 (7.7)
Occipital (%)	0 (0.0)	1 (16.7)	1 (7.7)
Multi-lobar (%)	0 (0.0)	2 (33.3)	2 (15.4)
Female sex (%)	43 (57.3)	29 (50.9)	72 (54.5)
**Surgical pathology**			
Mesial temporal sclerosis (MTS)	11 (14.7)	8 (14.0)	19 (14.4)
Malformations of cortical development (MCD)	31 (41.3)	27 (47.4)	58 (44.0)
Cryptogenic	5 (6.7)	3 (5.3)	8 (6.1)
MTS and MCD	19 (25.3)	9 (15.8)	28 (21.2)
Other	9 (12.0)	10 (17.5)	19 (14.4)

Characteristics of the cohort of drug-resistant epilepsy patients selected for each sub-group sequencing analysis. Age at surgery (years), epilepsy resection sub-type, and sex are reported. Cyrptotgenic pathologies were pathology reports with non-specific findings.

### Seizure outcomes

Engel scores (13) at last postoperative follow-up, date of surgery, last follow-up date, and date of seizure recurrence were collected under the Epilepsy Surgery Nomogram registry. Seizure freedom was defined as an Engel score of IA (completely free of seizures since surgery) or IB (free of disabling seizures since surgery). All patients with Engel scores of IIA—IVC were considered seizure recurrent post-operatively.

### Genetic variant selection and analyses

A goal of this study was to determine whether specific genetic features may aid in predicting seizure outcome after surgery. Candidate genes were selected with the goal of capturing several proposed mechanisms underlying post-operative seizure pathophysiology from prior literature. To conserve statistical power in this pilot study, we selected a total of 10 genes across genes that were either previously studied in the setting of surgical epilepsy seizure outcomes (*DEPDC5, NBEA, SCN1A*) (6), or studied as potential mechanisms for the cause or maintenance of the epileptogenic zone in focal epilepsy (6, 14–24). These mechanisms included: neuroinflammatory causes of epileptogenesis (*IL1A, C3*) (17), calcium regulation and its role in induction and maintenance of epilepsy (*CALHM1*) (17, 21), *mTOR* pathway regulation in focal epileptogenesis (*DEPDC5, PTEN*) (6, 15, 18, 20, 22), monogenic causes of focal epilepsy (*SCN1A, GABBRA1, LGI1*) (16, 17, 23), established genetic causes of epileptic encephalopathies (*NBEA*) (6, 18), and pharmaco-resistance mediating maintenance of seizures (*ABCB1*) (14, 23, 24). When studies identified associations with multiple genes in the same family, only a single gene was selected for inclusion in this study. The following 10 genes were selected for further analyses in this study: *SCN1A, NBEA, PTEN, GABRA1, LGL1, DEPDC5, IL1A, ABCB1, C3, CALHM1*.

We then identified all genetic variants (SNPs) detected within the genomic region of the 10 selected genes from variant call format (VCF) files from previously developed exome sequencing data (25) using PLINK v1.90 (26). Details regarding exome sequencing and processing can be found in the Supplementary Methods. SNPs with a minor allele frequency > 0.03 were selected. Using this approach, a total of 109 SNPs with minor allele frequencies (MAF) > 0.03 were extracted from these 10 genes and PLINK was used to test SNPs for associations with post-operative seizure outcome using logistic regression. PLINK was also used to perform a meta-analysis of SNPs by surgical subtype (81 temporal vs. 25 frontal and parietal patients combined). Because this is a pilot study, with very minimal prior evaluations of genetic variation and epilepsy surgery outcomes, we used a similar approach to Oliver et al. which selected 27 candidate genes for epileptic encephalopathy from a total of 179 genes using an empirical false discovery rate (FDR) of *P* < 0.25 as a threshold for statistical significance (18, 27). The statistical software, R v4.4 (28) and *ggplot2* (29) was used to visualize the proportion of alternate allele copies (0, 1, or 2) among seizure-free and recurrent patients.

#### Time-to-event analysis of seizure recurrence post-operatively

A Cox proportional hazards model was selected due to its ability to assess the statistical significance between individual SNPs and time-to-seizure recurrence. Additionally, using a Cox proportional hazards model allowed for the appropriate censoring of patients who were lost to follow-up, and also accounted for patients who may not have had as much follow-up time as other patients given our data is from is a real-world cohort. Time-to-seizure recurrence was calculated using the date of surgery and date of last-follow-up for at least 6 months post-surgery. Kaplan Meier curves were created using the SAS studio v3.7 (30) to depict the proportion of patients who remained seizure-free post-operatively with respect to number of alternate alleles.

A Fisher's exact test was performed on 14 anti-seizure medications (ASMs) and seizure outcome to test whether the SNP results were potentially confounded by a particular ASM. Results of this analysis can be found in Supplementary Figure 1.

#### Assessing single-nucleotide polymorphisms as predictors of seizure freedom

We investigated whether each SNP, identified in the time-to-event analysis, could improve discrimination of seizure outcome (seizure-free vs. recurrent) when combined with clinical variables. Each model incorporated the SNP and up to two clinical variables (limited to reduce the likelihood of overfitting) in a Cox proportional hazards model. Clinical predictors were selected from a set of candidate predictors [from nomogram previously described (8)] using Harrell's step-down procedure (31) to identify the two variables with the highest combined predictive accuracy for seizure-outcome: normal MRI and sex. C-statistics were further corrected for potential over-fitting using bootstrap resampling.

#### Evaluating SNPs as expression quantitative trait loci

SNPs can result in changes in amino acids (e.g., missense, nonsense mutations) or can cause no amino acid change (silent mutations). Among other mechanisms, SNPs can influence promoter activity, messenger RNA (mRNA) conformation (stability), and subcellular localization of mRNAs and proteins, resulting in phenotypic changes in disease states (32). In addition to influencing expression of the genes in which they are located, SNPs can also influence expression of distant genes. SNPs that modulate gene expression are known as expression quantitative trait loci (eQTLs). To assess whether the SNPs identified in our study may be eQTLs, we used the Genotype Tissue Expression database (GTEx). GTEx is a database containing data from multiple experiments from patients without known pathologies (33). SNPs associated with gene expression that had a *P* < 0.05 in GTEx were considered statistically significant.

### mRNA sequencing and analyses

Flash-frozen brain tissue samples were available from 135 patients at the time of resection. A complete description of mRNA sequencing sample collection, sequencing, and data processing for blood and tissue samples can be found in the Supplementary Methods. Briefly, after processing, normalized counts were summed by Ensembl gene ID using the R, *biomaRt* package v3.14 (34). Logistic regression models were used to test for associations between genes and binarized post-operative seizure outcomes (seizure-free vs. recurrent). As described above, a previously utilized exploratory FDR *P* < 0.25 was used as a threshold for statistical significance due to sample size limitations of this pilot study (18, 27).

### miRNA sequencing and analyses

Flash-frozen brain tissue samples were available from 132 patients for miRNA sequencing. A full description of miRNA sequencing sample collection, sequencing, and data processing can be found in the Supplementary Methods. Briefly, after miRNA libraries were sequenced and processed, we performed normalization of miRNA counts for both mature and hairpin miRNA using cpm normalization in *edgeR* (35) v3.26.8. Logistic regression was used to test for associations between each mature or hairpin miRNA and seizure outcome post-operatively using normalized miRNA. Consistent with the above analyses, a FDR *P* < 0.25 was considered to be the threshold for statistical significance.

### Pathway and gene enrichment analyses

Testing for individual associations between miRNAs or mRNAs and seizure outcomes can identify singular miRNAs or mRNAs associated with seizure freedom. Conversely, pathway analysis can identify coordinated effects of multiple RNAs (the miRNAs or mRNAs within a biological pathway) are associated with seizure outcomes. We performed pathway enrichment analysis with the Ingenuity Pathway Analysis (36) (IPA) software (Qiagen Bioinformatics) using *P-*values from the logistic regression models for miRNAs and mRNAs and seizure outcome (as described above). Pathways with FDR *P* < 0.05 were considered to be significantly enriched.

## Results

A total of 201 patients fulfilled our study criteria. One hundred and five patients (52.2%) were seizure-free post-surgery at time of last follow-up, and 96 patients (47.8 %) had recurrent seizures. Most (*N* = 167 patients, 83.1%) had temporal lobe epilepsy, and a smaller subset (*N* = 34, 17%) had extra-temporal epilepsy. Surgical pathologies of resected tissue included 93 patients with malformations of cortical development (MCD), 24 with mesial temporal sclerosis (MTS), 37 patients with both MCD and MTS, and 10 patients with cryptogenic epilepsy (non-specific findings on pathology). The mean age at time of surgery was 38.3 years. Approximately half were female (112 patients, 55.7%), and all patients self-identified as White. A full breakdown of resection tissue subtype and surgical pathologies is shown for seizure-free and recurrent patients in each analysis performed in this study in Table 1.

### SNPs in ABCB1 are associated with post-operative seizure outcomes

**Figure 1:**
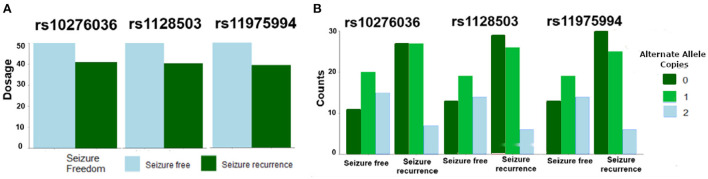
Single nucleotide polymorphisms (SNPs) in *ABCB1* were associated with likelihood of post-operative seizure recurrence. **(A)** An increase in allelic dosage of alternate alleles (sum of the number of alternate alleles) in the post-operative seizure-free subset of patients was observed compared to the seizure-recurrent subset for SNPs rs10276036, rs1128503, rs11975994. **(B)** Depicts the number of alternate allele copies by post-operative seizure outcome and by SNP.

Of the 109 SNPs across the 10 candidate genes evaluated in this study, only three SNPs located in *ABCB1* on chromosome 7 (rs10276036, rs11975994, and rs1128503) were associated with post-operative seizure freedom (FDR *P* = 0.24 for all three SNPs) (Table 2). All three SNPs were in linkage disequilibrium and were therefore highly correlated with each other. Patients who were seizure-free after surgery were more likely to have genotypes with the alternate alleles of these SNPs (Figures 1A,B). Despite small sample sizes, a stratified analysis based on resection tissue types (temporal cases and extratemporal cases) did not demonstrate heterogeneity between extratemporal cases compared to temporal cases; both trended toward seizure reduction post-operatively (Supplementary Figure 2).

**Table 2:** Genetic variants associated with seizure outcome.

**CHR**	**SNP ID**	**BP**	**Alternate allele**	**Reference allele**	***P***	**FDR-adjusted *P***
7	rs10276036	87,180,198	C	T	0.004846	0.239509
7	rs11975994	87,192,731	G	A	0.004965	0.239509
7	rs1128503	87,179,601	A	G	0.006592	0.239509

Number of SNPs that met the FDR-adjusted *P* < 0.25 with corresponding chromosome (CHR), single nucleotide polymorphism ID (SNP ID), base pair location (BP), reference allele, alternate allele, and non-adjusted *P*-value.

**Figure 2:**
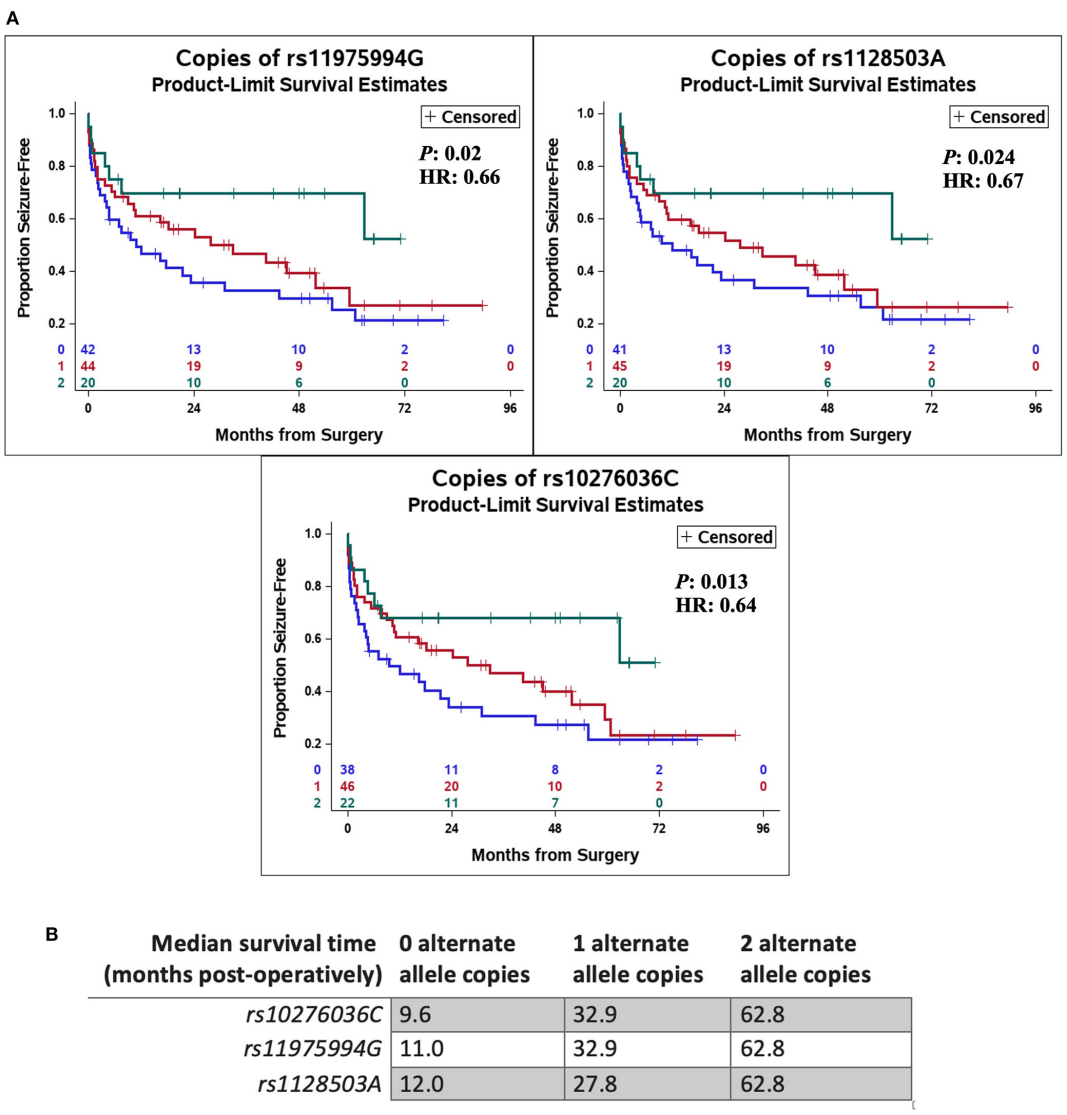
Genetic variation in ABCB1 was associated with delayed recurrence of post-operative seizures. Kaplan Meier curves using a Cox proportional hazards model of each independent single-nucleotide polymorphism (rs10276036, rs1128503, rs11975994) depict the proportion of patients who remain seizure free after surgery by number of alternate allele copies **(A)**. The green line represents having two alternate allele copies, the red line represents having one alternate allele copy, and the blue line depicts having zero alternate allele copies. Hazard ratios (HR) and *P*-values (*P*) are displayed on each Kaplan Meier curve. **(B)** Shows the median time-to-seizure in months post-operatively for each SNP and number of alternate allele copies.

In addition, SNPs in *ABCB1* (i.e., rs10276036, rs1128503, rs11975994*)* were also associated with the time to seizure recurrence post-operatively (*P* = 0.013, HR = 0.64; *P* = 0.024, HR = 0.67; and *P* = 0.02, HR = 0.66, respectively) (Figure 2). Patients with alternate alleles in *ABCB1* experienced longer periods of seizure-free time post-operatively (months from surgery) compared to patients without the alternate allele (Figure 2). The median time-to-seizure for patients with 2 alternate alleles for each of the three SNPs was 62.8 months post-surgery, compared to the median time-to-seizure for patients with 1 alternate allele (23.9 months for rs10276036, 32.9 months for rs11975994, and 27.8 months for rs1128503), and patients with 0 alternate alleles (9.6 months for rs10276036, 11 months for rs11975994, and 12 months for rs1128503) (Figure 2).

### Assessing SNPs as predictors of seizure freedom

Statistical significance does not necessarily indicate that the SNPs will discriminate outcomes sufficiently for prediction. After determining that the three SNPs in *ABCB1* were associated with seizure-freedom, we tested if these SNPs have potential to improve existing predictions of seizure freedom using the presence of a normal MRI and sex as predictors, which were identified based on a previous model as the best two predictors for seizure recurrence in this sample of patients (8). Table 3 demonstrates that the C-statistic (model prediction performance where 0.5 is no better than random chance) for normal MRI alone and sex alone were 0.58 and 0.54, respectively. Combining sex and normal MRI into a single model increased the C-statistic to 0.61. When SNPs were tested independently as predictors, the model performance was equivalent to using MRI alone, and performed slightly better than using sex alone, with a C-statistics ranging from 0.57 to 0.58 (Table 3). It is unsurprising that the predictive performance was similar for each of the three SNPs given that they are in linkage disequilibrium and therefore highly correlated with each other. Notably, the addition of each SNP to the combined model using normal MRI and sex, improved the accuracy of the model (C-statistic increased from 0.61 to 0.64; Table 3), indicating that these SNPs provide discriminative information that is independent of the other clinical factors.

**Table 3:** Performance of SNPs and clinical predictors in a cox proportional hazards model.

**Model**	**Raw C- statistic**	**Corrected C-statistic**	**HR**	***P***
**Best clinical variables without SNP**				
Normal MRI	0.58	0.58	1.75	**0.036**
Female sex	0.54	0.52	0.73	0.203
Normal MRI + Female sex	0.61	0.59		
**SNP alone**				
rs1128503A	0.57	0.57	0.67	**0.024**
rs10276036C	0.58	0.58	0.64	**0.013**
rs11975994G	0.58	0.57	0.66	**0.02**
**Best combined models**				
rs1128503A + sex, normal MRI	0.64	0.62	0.68	**0.035**
rs10276036C + sex, normal MRI	0.64	0.62	0.64	0.17
rs11975994G + sex, normal MRI	0.64	0.62	0.67	**0.030**

C-statistics are shown for independent clinical variables (presence of normal MRI or female sex) and for each SNP. C-statistics are also provided for combined models using each SNP alongside female sex and the presence of a normal MRI to predict post-operative seizure freedom. A corrected C- statistic was calculated using bootstrap resampling to correct for possible overfitting. Hazard ratios (HR) and *P*-values are shown for each Cox proportional hazards model.

### Evaluating SNPs as expression quantitative trait loci

**Figure 3:**
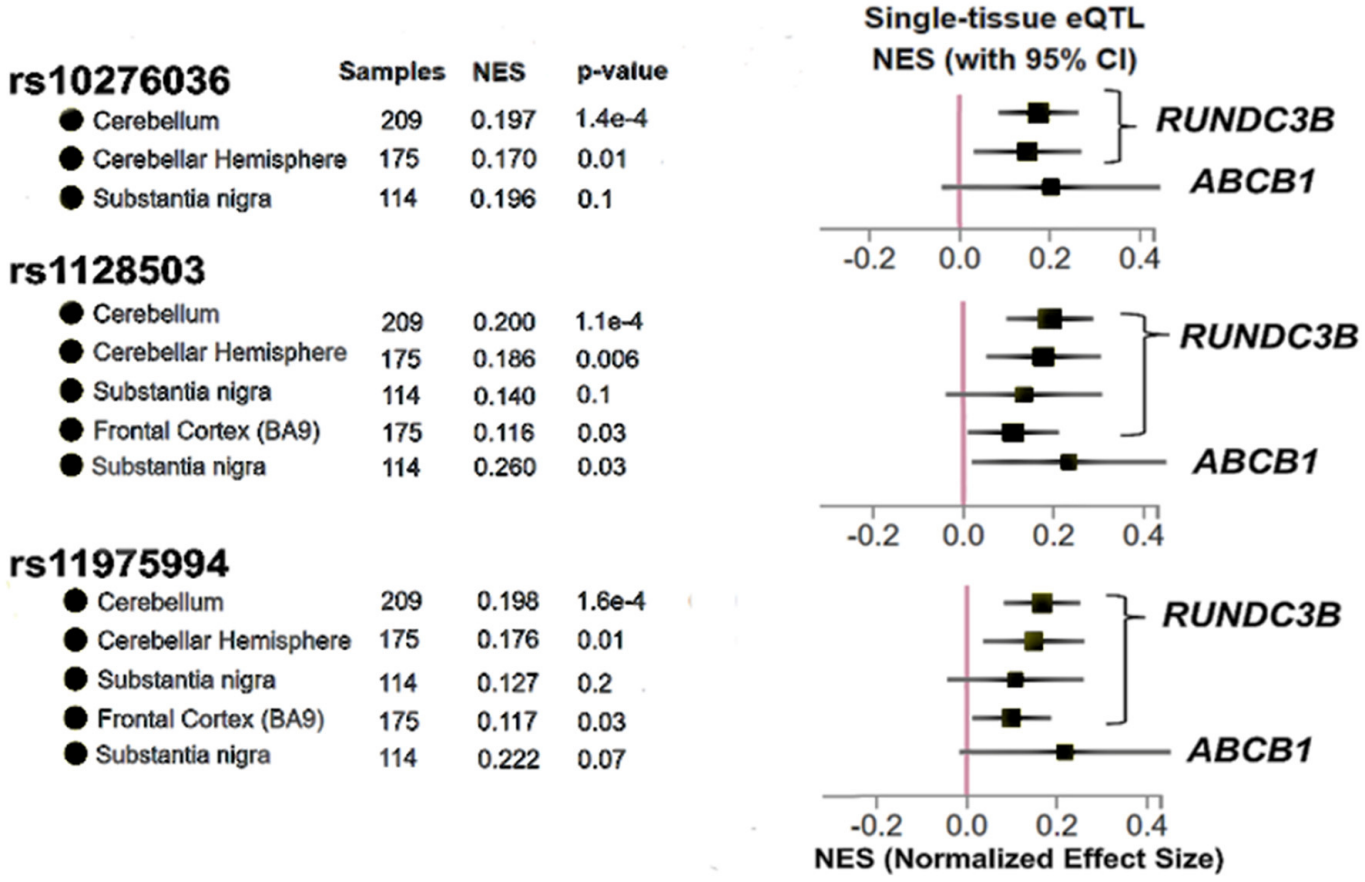
Results adapted from the Genotype Tissue Expression database (GTex) for the three SNPs identified in this study (rs10276036, rs1128503, rs11975994). The x-axis represents Normalized Effect Size (NES) where an increase in NES represents a SNP increasing expression of a particular gene, while a decrease in NES would represent a SNP modulating gene expression by decreasing expression. *P*-values shown for each brain region for each SNP are used to determine if a SNP is an expression quantitative trait locus shown for the gene shown on the right of the figure. *P* < 0.05 was used to determine significance.

In order to determine whether the three SNPs associated with post-operative seizure freedom have potential to affect gene expression in the brain, we used the GTEx database of tissue expression in patients without known pathologies. We found that SNPs rs10276036, rs1128503, and rs11975994 are eQTLs for modulating the expression of *RUNDC3B*, a protein coding gene expressed in the cerebellar hemispheres, cerebral cortex, hippocampus, and white matter (37) (Figure 3). Additionally, only rs11975994 was an eQTL for *ABCB1*, increasing its expression (Figure 3). These findings suggest that the protective effects of rs10276036, rs1128503, and rs11975994 on post-surgical seizure outcomes may be related to modulation of *RUNDC3B* or *ABCB1* expression.

### RNA sequencing and pathway enrichment analyses

**Figure 4:**
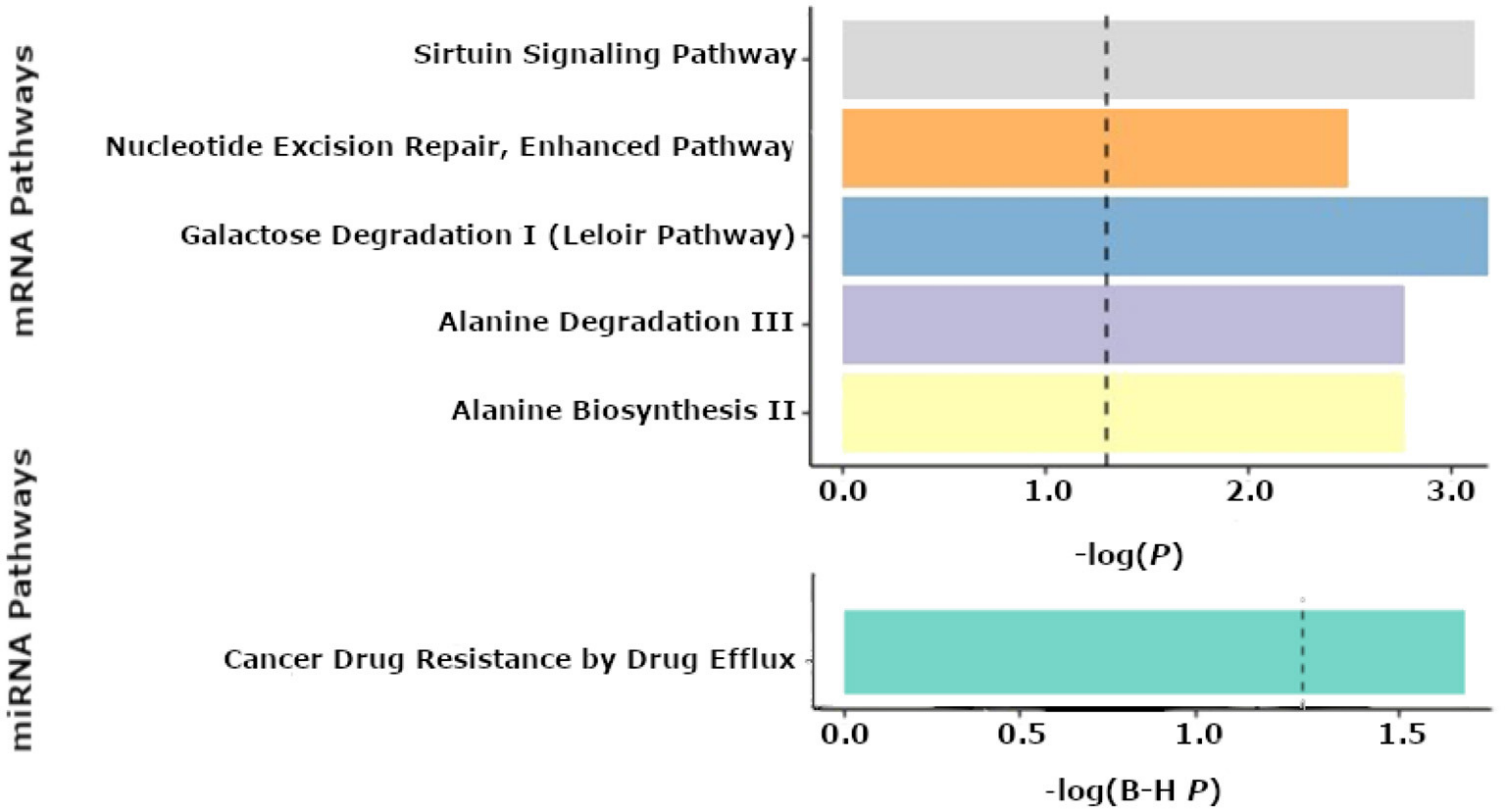
Pathway enrichment analyses from the Qiagen IPA tool. Results of enriched pathways from mRNAs when nominal *P*-values from the logistic regression testing for associations between mRNA and seizure outcome were filtered with a *P* < 0.05 threshold for all mRNAs prior to submitting mRNAs for pathway analysis in IPA. The *x*-axis shown represents –log(*P*-values), with a cutoff of 1.3 denoting a *P* < 0.05. The top 5 pathways are shown for mRNAs from tissue (top panel). Enriched microRNA (miRNA) pathways after nominal *p*-values from the logistic regression of miRNAs and seizure outcome were filtered with a *P* < 0.05 threshold prior to performing pathway analyses using IPA (bottom panel). The *x*-axis shown represents -log(FDR corrected *P*-values), with a cutoff of 1.3 representing a *P* < 0.05.

No statistically significant associations (FDR < 0.25) were found between individual mRNAs or miRNAs and seizure outcome. However, pathway analyses of miRNAs revealed “cancer drug resistance by drug efflux” to be an enriched pathway consisting of mir-154 and mir-379 expression (*P* = 0.02). In the tissue samples, metabolic pathways such as “alanine degradation and biosynthesis” (*P* = 0.002 for both pathways) as well as “galactose degradation” were significantly enriched (*P* = 0.001). mRNAs comprising the “Nucleotide excision repair” (*P* = 0.003) and “sirtuin signaling” (*P* = 0.001) pathways were also significantly associated with seizure-outcomes post-resection (Figure 4). The predicted networks describe functional relationships between miRNAs and genes based on a knowledgebase of predicted and experimentally observed relationships.

## Discussion

In this pilot study, we investigated multiple molecular modalities (SNPs, mRNAs, or non-coding RNAs) for association with post-operative seizure outcome. We had the dual goals of evaluating whether specific genomic features offer predictive potential for post-resection seizure freedom, and gaining some mechanistic understanding of surgical seizure outcomes. Our findings begin to help advance both questions.

### Augmenting the prediction of epilepsy surgery outcomes with genetics

Prediction of seizure outcomes following epilepsy surgery has improved over the past decade through predictive modeling approaches that integrate clinical, imaging, and electrophysiology data. Yet, the performance of clinical predictive models remains suboptimal warranting the need for further improvement. Imaging–based outcome prediction models uniformly suggest that an epileptic network that extends beyond the limits of a focal resection increase the risk of seizure recurrence. Although such models correlate structural or functional brain images with surgical outcomes, they do not provide etiological insight into the disorder. Furthermore, while a number of epilepsy risk genes have been previously identified (38), these genes are often associated with epilepsy phenotypes that do not typically present for consideration of epilepsy surgery (generalized or multifocal epilepsies or epileptic encephalopathies). Their significance for informing the mechanisms of seizure freedom or recurrence following resections for DRE is therefore unclear. In order to better understand *why* only certain patients have worse outcomes or early seizure recurrences post-operatively, studies are needed to test whether biological and genetic mechanisms can help bridge this gap in prediction.

We found that genetic variation in *ABCB1* was associated with a reduction in odds of seizure recurrence by 56, 53, and 54% for the SNPs rs10276036, rs1128503, and rs11975994, respectively (FDR *P* < 0.25). Our ability to fully explore the incremental contribution of these SNPs to other known predictors of post-operative seizure freedom was limited by the size of our cohort, but we still found that the predictive performance of these SNPs was equivalent to that of MRI, a heavily utilized diagnostic tool for selecting patients for epilepsy surgery and for the prediction of seizure outcomes. These results contribute toward the growing interest in identifying actionable biomarkers that could inform surgical decision-making.

### Linking *ABCB1* and *RUNDC3B* to seizure outcomes after surgery

*ABCB1* is a known multi-drug resistance gene that codes for P-glycoprotein (P-gp), an ATP-dependent efflux pump (39) thought to contribute to drug-resistance in epilepsy by preventing ASMs from reaching their target sites across the blood brain barrier (39). Yet, all patients in our cohort had DRE that necessitated brain surgery for epilepsy, and we did not identify any associations between seizure outcomes and ASM usage (Supplementary Figure 1). This suggests that variation in *ABCB1* may be related to surgical outcomes in a manner independent of its function of encoding a protein involved in drug efflux (40).

miRNAs are small non-coding RNAs that regulate gene-expression post-transcriptionally and have been shown to regulate a wide range of cellular functions including modulating cell growth in aging and neurodegenerative diseases (41). No mRNAs or miRNAs were found to be independently associated with post-operative seizure outcomes (FDR *P* > 0.05) in our cohort. Possible explanations include our limited sample size, or potential heterogeneity in the sampled tissue regions. However, we found that a pathway for cancer drug resistance by drug efflux involving hsa-mir-410 and hsa-mir-411 (FDR *P* = 0.017) was significantly enriched, further supporting the hypothesis that drug efflux pumps, such as P-gp, could play a mechanistic role in post-operative seizure-freedom.

The SNPs identified in this study are eQTLs for *RUNDC3B* or *ABCB1* expression in the brains of patients without neurological pathologies (the GTEx database consists of participants with no known pathologies) (33). While the function of *RUNDC3B* has not been well characterized, studies have shown that deletions greater than one megabase in *RUNDC3B* have been associated with sporadic epilepsy syndromes (42). Our results suggest that other mechanisms modulating *RUND3CB* may impact response to epilepsy treatments, although no significant associations between mRNA expression of *ABCB1* or *RUNDC3B* and seizure outcome were observed here (FDR *P* > 0.05). This may be because the eQTLs found in GTEx were for the substantia nigra and cerebellum only, while our analyses investigated cortical tissue mostly within the temporal lobe, with a smaller subset of tissue collected from frontal, parietal and occipital lobes. Alternatively, the mechanism by which *ABCB1* or *RUNDC3B* influences post-operative seizure freedom may not be modulated by gene expression, but rather functional changes to the protein or other mechanisms might play a role.

### Beyond drug-resistance, hypothesizing possible mechanisms of *ABCB1*'*s* contribution to seizure outcomes after surgery

Prior experimental work has linked *ABCB1* activation and expression of its protein, P-gp, with cell hypoxia akin to what occurs “locally” in the surgical bed during brain surgery for epilepsy (43). Wang et al. showed the expression of hypoxia-inducible factor-1 alpha (HIF-1α) was positively correlated with the expression of P-gp in patients with refractory epilepsy and expressions of both (HIF-1α and P-gp) at mRNA and protein levels was reduced after transfection with small interfering (siRNA) targeting HIF-1α (43). The authors concluded that HIF-1α may be involved in the upregulation of P-gp in refractory epilepsy through inducement of P-gp expression and that activation of the HIF-1α/P-gp pathway is one hypothesis to explain the pathogenesis of refractory epilepsy. Similarly, others have observed that ABC-transporters, which can be constitutively expressed in normal progenitor or cancer stem-cells, are overexpressed in malformations of cortical development (MCD) and brain tumors (44). Irrespective of its role in drug-resistance, P-gp also plays a role in membrane depolarization (44), and it is thought that constitutive P-gp overexpression in MCD and brain tumors may explain their epileptogenic properties, and the conclusion was that ABC-transporters could help to better identify abnormal progenitor cells and serve as a biomarker of risk for seizure relapse after epilepsy surgery (44).

## Limitations and conclusions

As with any study, there were important limitations that should be considered. Not all sample types were available on all patients resulting in some analytical platforms having smaller sample sizes than others. The inclusion of extratemporal tissue types may have impacted the analysis of mRNA tissue expression and larger cohorts will be required to understand the tissue-specific relationships with mRNA expression and surgical outcomes (Supplementary Figure 2). In addition, all patients available in this study self-identified as White, and whether these results are generalizable to more diverse populations will need additional research. Notably, SNPs rs10276036, rs1128503, and rs11975994 have higher MAF in other ethnicities, such as those of African ancestry (MAF = 0.8–0.86) (45). Furthermore, the sample size available for this pilot study limited the number of genes we could investigate. Therefore, we focused our analysis on genes we hypothesized would be most relevant to this clinical context (focality of epilepsy, and brain injury from surgical resection) as well as focusing on genes across multiple mechanisms mediating epileptogenesis post-operatively. However, we acknowledge that our selected gene list does not represent an exhaustive list of all genes that should be explored; especially as it pertains to other multi-drug resistant (MDR) genes given our findings with *ABCB1*. Despite these limitations, this study suggests that genomic and molecular features should be further investigated in larger cohorts that would allow for the interrogation of a more complete gene list involving other known MDR genes to better evaluate the role between RNAs and SNPs and epilepsy surgical outcomes. More comprehensive and definitive studies are needed with larger sample sizes to confirm these findings, identify additional novel associations and provide opportunities for improved risk prediction tools that can be incorporated into the provision of care for these patients.

In conclusion, a multi-modal analysis indicated the putative role of drug efflux pathways, and in particular genomic variation in *ABCB1*, as potential modifiers of post-surgical seizure freedom in patients with epilepsy. In addition, genetic variation in *ABCB1* was identified as an independent predictor of post-surgical seizure freedom, indicating the need for additional research to determine whether incorporating these SNPs as biomarkers into existing clinical prediction tools is warranted.

## Data availability statement

The datasets presented in this article are not readily available because of ethical and privacy restrictions. Requests to access the datasets should be directed to LJ, jehil@ccf.org.

## Ethics statement

The studies involving human participants were reviewed and approved by Cleveland Clinic Institutional Review Board. Written informed consent for participation was not required for this study in accordance with the national legislation and the institutional requirements.

## Author contributions

SL, LJ, and DR wrote and revised the initial manuscript. DL, OH, PB, and SL performed pertinent analyses and data processing for analyses presented in this study. RB, MM-S, and DV assisted with the obtaining and storage of data from their respective databases. All authors were involved in the interpretation of results, preparation, and review of this manuscript. All authors contributed to the article and approved the submitted version.

## Funding

This study was supported by the Cleveland Clinic Lerner Research Institute Center of Excellence for Epilepsy and Co-morbidities Research, the Stanley Center for Psychiatric Research at Broad Institute, the Clinical and Translational Science Collaborative of Cleveland (KL2TR000440, UL1TR000439), and the Charles Shor Epilepsy Center, Cleveland Clinic. This work was additionally supported with funding from NINDS/NIH 1R01NS097719 and whole exome sequencing was funded by philanthropic funds from the Broad Institute and Mark Daly.

## Conflict of interest

Author DR has an equity stake in Clarified Precision Medicine, LLC. DR has received research support from Novo Nordisk, consulting honoraria from Interpares Biomedicine and Pharmazaam, LLC. Author CE is the Sondra J. and Stephen R. Hardis Endowed Chair of Cancer Genomic Medicine at the Cleveland Clinic. The remaining authors declare that the research was conducted in the absence of any commercial or financial relationships that could be construed as a potential conflict of interest.

## Publisher's note

All claims expressed in this article are solely those of the authors and do not necessarily represent those of their affiliated organizations, or those of the publisher, the editors and the reviewers. Any product that may be evaluated in this article, or claim that may be made by its manufacturer, is not guaranteed or endorsed by the publisher.
