# Supplementary material for: Genetic and molecular features of seizure-freedom following surgical resections for focal epilepsy: A pilot study
Source: Front Neurol. 2022 Sep 16;13:942643. doi: 10.3389/fneur.2022.942643 (PMC9524264; doi:10.3389/fneur.2022.942643)
Supplement: Supplementary file 1 [file Data_Sheet_1.docx]

**Supplementary Material**

**Supplemental Methods**

*Whole exome Sequencing & Processing*

WES was performed by the Broad Institute of Harvard and the Massachusetts Institute of Technology (Cambridge, MA)^1^. Exome capture was performed on blood samples from 106 patients using Illumina Nextera Rapid Capture Exomes enrichment kit. Paired-end reads (151 bp) were sequenced on the Illumina HiSeq 4,000. BAM files were then generated and aligned to the GRCh37 human reference genome before undergoing quality control measures using the Picard data-processing pipeline (https://broadinstitute.github.io/picard/)^2^. SNPs were called using the genomic analysis toolkit (GATK) with the resulting data stored in variant call format (VCF)^3^. VCF files were than used to extract SNPs of interest for this study in our cohort of 106 patients.

*mRNA Sequencing & Processing*

Brain tissue was collected from 135 patients at the time of resection and homogenized in 1 mL Trizol (Life Technologies) and TissueLyser II (Qiagen). Blood samples were collected from 74 patients. Total RNA was extracted from tissue or blood using Trizol and the Ambion PureLink RNA Mini Kit (Life Technologies) for RNA cleanup and DNase treatment. RNeasy Lipid Tissue Kit (Qiagen) was used to extract RNA (total RNA and small RNAs). RNA quality was assessed using a 2100 Bioanalyzer (Agilent Technologies) with a RNA integrity number (RIN) score cutoff of 7.

TruSeq Stranded Total RNA kit (Illumina) was used for library preparation and sequencing was performed using the Illumina HiSeq 2000 platform (100 base pairs, paired-end) at the Genomics Core of the Lerner Research Institute at Cleveland Clinic (Cleveland, OH). Quality control, read filtering, and adapter trimming were performed using fastp^4^, and transcriptome quantification was performed using Salmon v0.14.1^5^ to align to the hg38 human genome using annotations from Gencode^6^. Raw counts were obtained and merged with tximport^7^ and saved as count and transcript length files as well as length scaled TPM files.

Versioned Ensembl gene IDs from TPM-normalized counts for tissue (n = 135) and blood mRNA (n=74) were converted to their corresponding HUGO Gene Nomenclature Committee (HGNC) gene names using biomaRt^8,9^. To reduce the overall number of genes tested for analyses, normalized counts were summed up for each gene as multiple Ensembl IDs mapped to the same gene,

*microRNA Sequencing & Processing*

The NEXTFLEX Small RNA-Seq Kit v3 (PerkinElmer) was used to prepare sequencing libraries for miRNA. Libraries were sequenced using the Illumina NovaSeq 6000 platform (100 base pairs, single-end) at the Genomics Core of the Lerner Research Institute at Cleveland Clinic (Cleveland, OH). Raw FASTQ files underwent quality read assessments using FASTQC v.0.11.8^10^. The nf-core smrnaseq workflow revision v1.0.0^11^ was used including quality trimming with Trim Galore v0.6.3^12^ and alignment to miRbase was performed using Bowtie 1 v1.2.2^13^ to mature miRNA sequences or stem-loop miRNA sequences (hairpin miRNA). Aligned BAM files were sorted, and counts were then calculated using *idxstats* from SAMtools v1.9^14^.

*Antiseizure Medications vs. Seizure Outcome*

Given the known association between *ABCB1*, its protein product, P-glycoprotein, and ASMs, a Fisher’s exact test was used to determine if any associations existed between any of the 14 ASMs (lamotrigine, oxcarbazepine, topiramate, levetiracetam, lacosamide, clonazepam, phenytoin, valproate, eslicarbazepine, phenobarbital, zonisamide, carbamazepine, lorazepam and ezogabine) used by our cohort and seizure outcome that could potentially confound the results of our SNP analyses. A threshold of FDR P < .05 was used to determine statistical significance. Since the purpose of this analysis was to rule out confounding due to the presence of a particular ASM that may be affected by P-glycoprotein, we did not assess combinations of ASMs, dosage of ASMs, or blood levels of ASMs in this study.

**Supplemental Results**


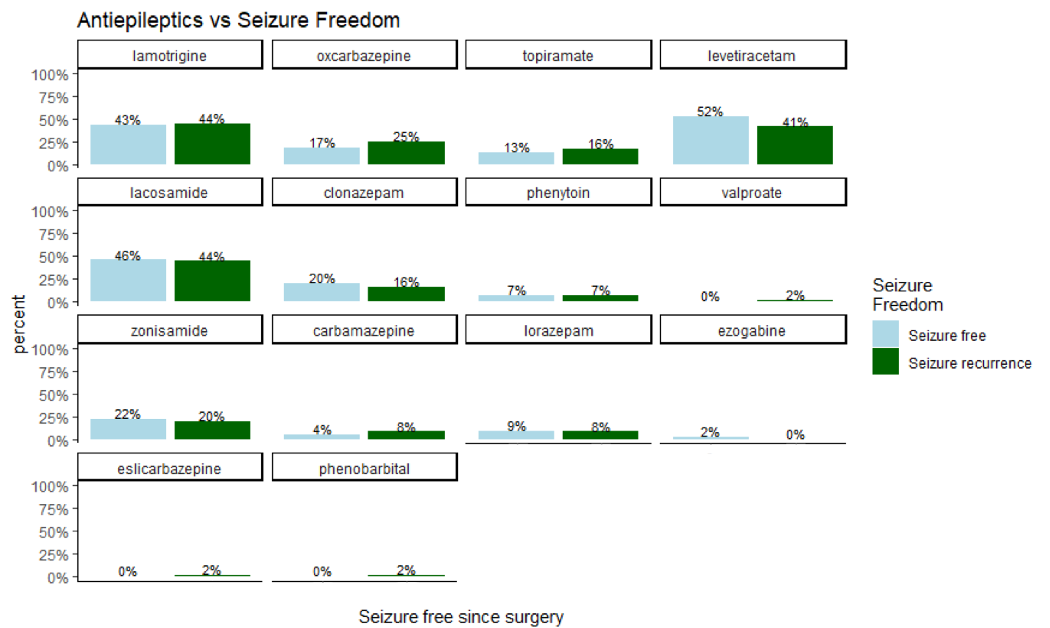
*Figure 1: Anti-seizure medication (ASMs) versus post-operative seizure outcome analysis of the subset of patients that underwent whole-exome sequencing (N = 106). A Fisher’s exact test was used to determine if any of the 14 ASMs used by this cohort were significantly associated with seizure outcome (P < .05). Light blue bars depict seizure- free patients, while dark green bars represent seizure-recurrent patients.*


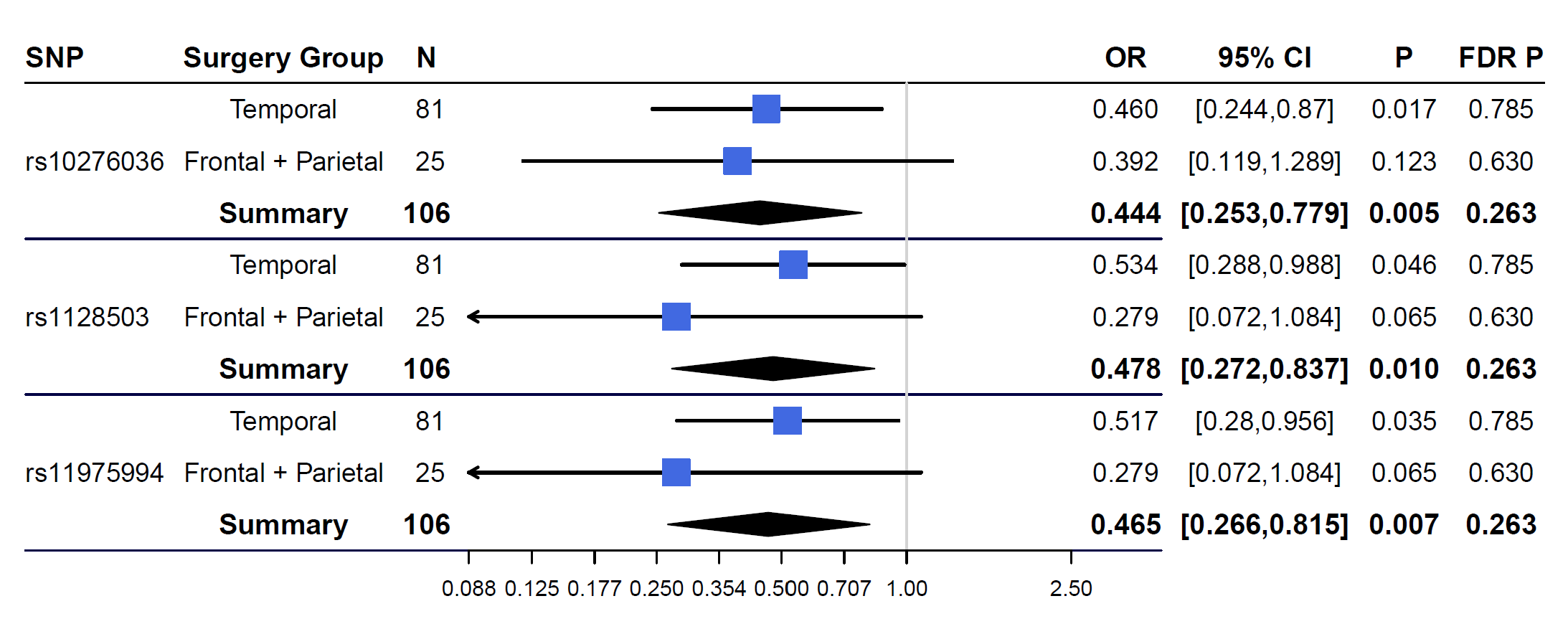
*Figure 2: Meta-analysis of SNPs by surgical subtype. X-axis represents the odds ratio (OR) of post-operative seizure recurrence, with an odds ratio of 1.00 representing no increased or decreased odds of seizure recurrence. For each SNP, the number of samples within each surgical subgroup is given (N), OR, 95% confidence interval (95% CI), and P values are shown. A significance threshold of P < .05 was used.*

Patients with alternate alleles of the three *ABCB1* SNPs within the temporal resection sub-group, had a significant reduction in the odds of seizure recurrence post-operatively. Patients with the C allele for rs10276036 were 64% less likely to have post-operative seizure recurrence (OR=0.46, 95% CI: [0.24-0.87], *P*=.017) while patients with the A allele for rs1128503 were 47% less likely to have post-operative seizure recurrence (OR=0.53, 95% CI: [0.288-0.99], *P* = .046). Patients with the G allele for rs11975994 were 48% less likely to have seizure recurrence post-operatively (OR = 0.52, 95% CI: [0.28-0.96], P = .035) (Figure 2). Patients in the combined frontal and parietal resection sub-group had a reduction in the odds of seizure recurrence, however this reduction in the odds ratio failed to reach statistical significance for rs10276036 (P=.12), rs1128503 (P=.06), and rs11975994 (P=.06). Patients in the meta-analysis (all patients across surgery groups) had a significant reduction in odds of seizure recurrence for all three SNPs: rs10276036 (OR=0.44,95% CI: [0.25-0.78], P=.005), rs1128503 (OR=0.48,95% CI: [0.27-0.84], P=.009), and rs11975994 (OR=0.47,95% CI: [0.26-0.82], P=.007) (Figure 2). These results indicate that SNPs rs10276036, rs1128503, and rs11975994 are associated with post-operative seizure outcome and may be candidates for predictors of surgical outcomes.

**References:**

1. Leu C, Bautista JF, Sudarsanam M, Niestroj LM, Stefanski A, Ferguson L, et al. Neurological disorder-associated genetic variants in individuals with psychogenic nonepileptic seizures. *Sci Rep*. 2020;10(1):15205. doi:10.1038/s41598-020-72101-8

2. *Picard*. Broad Institute Accessed September 16, 2020. http://broadinstitute.github.io/picard

3. McKenna A, Hanna M, Banks E, Sivachenko A, Cibulskis K, Kernytsky A, et al. The Genome Analysis Toolkit: A MapReduce framework for analyzing next-generation DNA sequencing data. *Genome Res*. 2010;20(9):1297-1303. doi:10.1101/gr.107524.110

4. Chen S, Zhou Y, Chen Y, Gu J. fastp: an ultra-fast all-in-one FASTQ preprocessor. *Bioinformatics*. 2018;34(17):i884-i890. doi:10.1093/bioinformatics/bty560

5. Patro R, Duggal G, Love MI, Irizarry RA, Kingsford C. Salmon provides fast and bias-aware quantification of transcript expression. *Nat Methods*. 2017;14(4):417-419. doi:10.1038/nmeth.4197

6. Frankish A, Diekhans M, Ferreira AM, Johnson R, Jungreis I, Loveland J, et al. GENCODE reference annotation for the human and mouse genomes. *Nucleic Acids Res*. 2019;47(D1):D766-D773. doi:10.1093/nar/gky955

7. Soneson C, Love MI, Robinson MD. Differential analyses for RNA-seq: transcript-level estimates improve gene-level inferences. *F1000Research*. 2015;4:1521. doi:10.12688/f1000research.7563.1

8. Durinck S, Spellman PT, Birney E, Huber W. Mapping identifiers for the integration of genomic datasets with the R/Bioconductor package biomaRt. *Nat Protoc*. 2009;4(8):1184-1191. doi:10.1038/nprot.2009.97

9. Durinck S, Moreau Y, Kasprzyk A, Davis S, De Moor B, Brazma A, et al. BioMart and Bioconductor: a powerful link between biological databases and microarray data analysis. *Bioinformatics*. 2005;21(16):3439-3440. doi:10.1093/bioinformatics/bti525

10. Andrews S. *FASTQC. A Quality Control Tool for High Throughput Sequence Data*.; 2010. http://www.bioinformatics.babraham.ac.uk/projects/fastqc/

11. Ewels PA, Peltzer A, Fillinger S, Alneberg J, Patel H, Wilm A, et al. Nf-Core*: Community Curated Bioinformatics Pipelines*. Bioinformatics; 2019. doi:10.1101/610741

12. Krueger F. *Trim Galore*. Accessed September 16, 2020. https://github.com/FelixKrueger/TrimGalore

13. Langmead B, Salzberg SL. Fast gapped-read alignment with Bowtie 2. *Nat Methods*. 2012;9(4):357-359. doi:10.1038/nmeth.1923

14. Li H, Handsaker B, Wysoker A, Fennell T, Ruan J, Homer N, et al. The Sequence Alignment/Map format and SAMtools. *Bioinformatics*. 2009;25(16):2078-2079. doi:10.1093/bioinformatics/btp352
